# Kisspeptin as a Precision Biomarker in Personalized Pharmacy: Implications for Individualized Monitoring of Early Pregnancy Viability

**DOI:** 10.3390/pharmacy14030084

**Published:** 2026-06-09

**Authors:** Ani Paunova, Angelina Mollova-Kyosebekirova, Maria Koleva, Ekaterina Uchikova, Nikoleta Parahuleva

**Affiliations:** 1 Department of Obstetrics and Gynecology, Medical Faculty, Medical University of Plovdiv, 4002 Plovdiv, Bulgaria; ani.paunova@mu-plovdiv.bg (A.P.); ekaterina.uchikova@mu-plovdiv.bg (E.U.); nikoleta.parahuleva@mu-plovdiv.bg (N.P.); 2 Clinic of Obstetrics and Gynecology, St. George University Hospital, 4002 Plovdiv, Bulgaria; 3 Department of General and Clinical Pathology, Medical Faculty, Medical University of Plovdiv, 4002 Plovdiv, Bulgaria; mariya.koleva@mu-plovdiv.bg

**Keywords:** kisspeptin, precision medicine, individualized monitoring, early pregnancy loss, miscarriage, placental biomarkers, immunohistochemistry, pharmaceutical care

## Abstract

Background: Precision medicine aims to improve early, individualized risk stratification using biologically relevant biomarkers. In early pregnancy, markers reflecting placental function remain limited. Kisspeptin, a placentally derived peptide that rises during normal gestation, has emerged as a potential indicator of pregnancy viability. Objectives: We aimed to evaluate evidence on maternal serum kisspeptin levels and placental KISS1/KISS1R expression in early pregnancy loss, and to assess its potential relevance as a precision biomarker within personalized pharmacy and individualized monitoring frameworks. Methods: A systematic search of PubMed, Scopus, Web of Science, and Google Scholar (up to 2025) was conducted according to the PRISMA 2020 guidelines. Studies assessing circulating kisspeptin and/or placental expression in early pregnancy loss versus viable pregnancies were included. A formal meta-analysis was not performed due to substantial heterogeneity in study design, biological material, assay methods, gestational age, and outcome reporting formats. Under these conditions, quantitative pooling was considered methodologically inappropriate; therefore, qualitative synthesis was performed. Results: Six studies met the inclusion criteria. Most reported significantly lower maternal serum kisspeptin levels in early pregnancy loss, with good discriminatory accuracy. Immunohistochemical analyses showed reduced placental and choriodecidual KISS1/KISS1R expression in miscarriage and recurrent pregnancy loss, indicating disrupted local signaling. Concordant systemic and tissue findings suggest that circulating kisspeptin reflects placental dysfunction. Conclusions: Kisspeptin appears to be a promising precision biomarker for monitoring early pregnancy viability and supporting individualized pharmaceutical care. Standardized assays and large prospective studies are needed before routine clinical implementation.

## 1. Introduction

Precision medicine has fundamentally reshaped contemporary healthcare by enabling risk assessment, monitoring, and therapeutic decision-making tailored to individual biological characteristics rather than population averages. Within this paradigm, personalized pharmacy plays an increasingly important role by translating biomarker-driven evidence into individualized monitoring strategies, pharmaceutical care, and interdisciplinary clinical support. In early pregnancy, however, current biochemical markers remain limited in their ability to reflect underlying placental function and to support individualized risk stratification. Kisspeptin, a placentally derived peptide encoded by the KISS1 gene, rises markedly during normal gestation and has emerged as a biologically specific indicator of placental viability. Growing evidence suggests that reduced circulating kisspeptin levels and altered placental kisspeptin signaling are associated with early pregnancy loss, positioning kisspeptin as a promising precision biomarker with direct relevance to personalized pharmacy and individualized monitoring of early pregnancy viability.

Kisspeptin, a peptide product of the KISS1 gene, has been recognized as a critical regulator of reproductive function. While it is well known for its role in stimulating gonadotropin-releasing hormone (GnRH) secretion, recent evidence highlights its involvement in peripheral reproductive processes, including trophoblast activity and placental development. During normal pregnancy, circulating kisspeptin levels rise markedly, primarily due to placental synthesis, indicating that maternal serum concentrations may serve as a marker of placental function and pregnancy viability [1]. Several observational studies have linked low first trimester kisspeptin levels with an increased risk of miscarriage [2]. For example, one large prospective cohort involving nearly 1000 asymptomatic pregnant women found that plasma kisspeptin measured at the booking antenatal visit was approximately 60% lower in those who later miscarried, compared to women with ongoing pregnancies. Some individual studies suggested that kisspeptin may offer favorable discriminatory performance compared with hCG; however, this observation has not yet been consistently validated across standardized cohorts [3]. In a case–control study, serum kisspeptin-54 concentrations were shown to discriminate between viable intrauterine pregnancies and spontaneous abortions, demonstrating its potential utility as a non-invasive biomarker [4]. At the tissue level, immunohistochemical and molecular studies have provided further insight. Colak et al. reported significantly decreased placental KISS1 protein expression in first-trimester samples from spontaneous abortions, suggesting impaired local kisspeptin signaling in failed pregnancies [2]. Babwah et al. also described that both KISS1 and its receptor KISS1R are downregulated in miscarriage placentas compared with uncomplicated pregnancies, highlighting a possible mechanistic link to defective trophoblast invasion [5]. Conversely, in certain gestational disorders such as preeclampsia, a paradoxical pattern is observed: while circulating kisspeptin is reduced, placental expression may be elevated, suggesting complex regulation of kisspeptin at the maternal–fetal interface [6]. Furthermore, the role of kisspeptin in trophoblast biology has been substantiated by functional studies. Li et al. demonstrated that in an animal model of recurrent spontaneous abortion (RSA), downregulation of KISS1 and its receptor GPR54 (KISS1R) was associated with reduced trophoblast invasiveness, implicating kisspeptin as a negative regulator of trophoblast migration and invasion in a dose-dependent manner [7]. More recently, immunohistochemical analyses in recurrent pregnancy loss (RPL) cases revealed altered KISS1/KISS1R expression in the choriodecidual tissue, further supporting a local, tissue-level disruption of kisspeptin signaling in pathological pregnancies [8].

Recent evidence further supports the role of kisspeptin in placental biology, trophoblast invasion, and early pregnancy viability. Experimental studies demonstrated that kisspeptin acts as a physiological regulator of trophoblast invasion and placental implantation [9,10] while altered placental expression of KISS1/KISS1R has been associated with pathological pregnancies such as preeclampsia [11]. At the same time, the growing field of precision medicine emphasizes the importance of biologically specific biomarkers for individualized monitoring and risk assessment [12,13]. In this context, pregnancy-specific biomarkers such as kisspeptin have attracted increasing interest because of their potential role in predicting early pregnancy complications and improving personalized clinical care [14].

Early pregnancy loss affects approximately 10–20% of clinically recognized pregnancies and remains a major challenge in obstetric care, with limited reliable biomarkers reflecting early placental dysfunction. Increasing evidence suggests that abnormal placentation and impaired trophoblast invasion, rather than embryonic factors alone, play a central role in the pathogenesis of early miscarriage.

Taken together, these findings position kisspeptin not only as a key physiological regulator of placentation but also as a promising biomarker of early pregnancy viability. However, despite growing interest, the precise relationship between maternal serum kisspeptin levels, placental expression assessed by immunohistochemistry, and early pregnancy loss remains incompletely characterized. Heterogeneity in study design, gestational age at sampling, analytical techniques, and tissue assessment has contributed to inconsistent findings across the literature.

Against this background, the aim of the present systematic review was to synthesize and critically evaluate available evidence on maternal serum kisspeptin levels and placental *KISS1/KISS1R* expression in early pregnancy loss. By integrating findings from circulating biomarker studies and placental immunohistochemical analyses, this review seeks to clarify the role of kisspeptin as a precision biomarker and to discuss its relevance for personalized pharmacy practice and individualized monitoring of early pregnancy viability.

Within the scope of personalized pharmacy, biomarkers are relevant not only when they directly guide medication selection, but also when they support individualized monitoring, interpretation of patient-specific laboratory profiles, risk communication, and multidisciplinary pharmaceutical care. In early pregnancy, where medication-related counseling and follow-up planning may be required, biologically meaningful markers of placental viability may therefore have translational relevance for future pharmacist-supported care models. Kisspeptin has attracted increasing interest in this context, as reduced circulating levels have been associated with miscarriage risk in several human studies [3,4], while broader evidence highlights its role in placental biology and pregnancy-related disorders [6]. However, available data remain heterogeneous and limited in number, and further validation is required before routine clinical application.

## 2. Materials and Methods

### 2.1. Search Strategy and Information Sources

This systematic review was conducted and reported in accordance with the Preferred Reporting Items for Systematic Reviews and Meta-Analyses (PRISMA 2020) guidelines [15]. A systematic literature search was performed in PubMed/MEDLINE, Scopus, Web of Science, and Google Scholar to identify relevant studies published up to 31 December 2025.

The search strategy combined Medical Subject Headings (MeSH) and free-text terms related to kisspeptin and early pregnancy outcomes. The following keywords were used individually and in Boolean combinations: “kisspeptin”, “KISS1”, “KISS1R”, “GPR54”, “kisspeptin-54”, “early pregnancy loss”, “miscarriage”, “spontaneous abortion”, “recurrent pregnancy loss”, “recurrent spontaneous abortion”, “pregnancy viability”, “viable pregnancy”, “non-viable pregnancy”, “placenta”, “placental tissue”, “choriodecidua”, “trophoblast”, “trophoblast invasion”, “immunohistochemistry”, “IHC”, “serum”, “plasma”, and “biomarker”.

The core search strategy was based on the following combination:

(“kisspeptin” OR “KISS1” OR “KISS1R” OR “GPR54” OR “kisspeptin-54”)

And

(“early pregnancy loss” OR “miscarriage” OR “spontaneous abortion” OR “recurrent pregnancy loss” OR “recurrent spontaneous abortion” OR “pregnancy viability”)

And

(“placenta” OR “placental tissue” OR “choriodecidua” OR “trophoblast” OR “immunohistochemistry” OR “serum” OR “plasma” OR “biomarker”).

Equivalent search strategies were adapted for Scopus and Web of Science. Google Scholar was searched using simplified keyword combinations, including “kisspeptin miscarriage”, “kisspeptin early pregnancy loss”, “KISS1 placental expression miscarriage”, “KISS1R recurrent pregnancy loss”, and “kisspeptin trophoblast invasion”.

To minimize publication bias, reference lists of eligible studies and relevant review articles were manually screened. Gray literature sources identified through Google Scholar were evaluated; however, conference abstracts, editorials, narrative reviews, unpublished theses, and non-peer-reviewed materials were excluded because they did not provide sufficient methodological detail or extractable outcome data.

The database search identified 284 records: PubMed/MEDLINE (*n* = 74), Scopus (*n* = 96), Web of Science (*n* = 68), and Google Scholar (*n* = 46). After removal of duplicate records (*n* = 88), 196 unique records remained for title and abstract screening.

### 2.2. Study Selection and Review Process

Study selection was conducted according to PRISMA 2020 recommendations. Two reviewers independently screened titles and abstracts for potential eligibility. Full-text articles considered potentially relevant were subsequently assessed against the predefined inclusion and exclusion criteria. Disagreements were resolved through discussion and consensus among the authors.

The literature search and initial screening were performed by A.P. and A.M.K. Data extraction and evidence synthesis were conducted by A.P. and M.K. Methodological supervision, eligibility verification, and critical review of the manuscript were performed by N.P. and E.U. All authors participated in interpretation of findings and approved the final manuscript.

**Figure 1:**
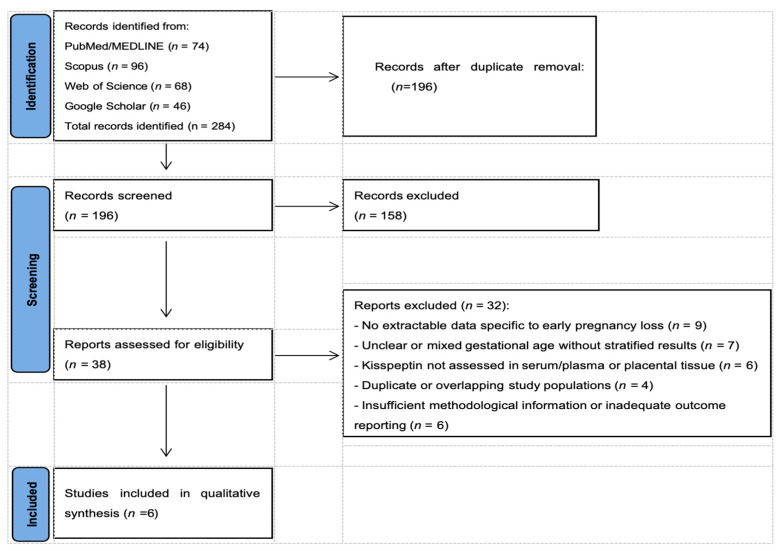
PRISMA 2020 flow diagram of the study selection process.

The initial screening process resulted in 196 records. A total of 158 records were excluded based on title and abstract review. Thirty-eight full-text articles were assessed for eligibility. Of these, 32 reports were excluded due to lack of extractable data specific to early pregnancy loss, unclear gestational age, absence of kisspeptin assessment in serum/plasma or placental tissue, duplicate or overlapping populations, or insufficient methodological information. Ultimately, six studies met the inclusion criteria and were included in the qualitative synthesis. The study selection process is presented in Figure 1.

The diagram illustrates identification, screening, eligibility assessment, and final inclusion of studies evaluating circulating and/or placental kisspeptin in relation to early pregnancy loss.

### 2.3. Inclusion Criteria

Studies were eligible for inclusion if they met the following criteria:

- Observational cohort studies (prospective or retrospective), case–control studies, cross-sectional studies, or diagnostic accuracy studies;
- Women with intrauterine pregnancy in early gestation, including groups with early pregnancy loss (EPL) or recurrent pregnancy loss (RPL);
- Viable ongoing early pregnancy confirmed clinically and/or by ultrasound as a comparator group;
- Assessment of circulating kisspeptin levels (serum or plasma, any isoform or analytical method) and/or placental or choriodecidual expression of KISS1 and/or KISS1R;
- Reporting extractable comparative data regarding pregnancy viability or early pregnancy loss.

### 2.4. Exclusion Criteria

Studies were excluded if they:

- Were non-human or exclusively in vitro studies;
- Did not provide extractable data specific to early pregnancy loss;
- Reported unclear or mixed gestational age without stratified analyses;
- Did not assess kisspeptin-related biomarkers in serum, plasma, placental tissue, or choriodecidual tissue;
- Were case reports, small case series, editorials, narrative reviews, letters, conference abstracts, or non-peer-reviewed publications;
- Represented duplicate publications or overlapping study populations.

### 2.5. Data Extraction

Data extraction was performed using a predefined standardized form. The following information was collected from each eligible study: author, year of publication, country, study design, study population, sample size, gestational age, biological material analyzed, biomarker assessed, analytical method, outcome measures, main findings, statistical significance, diagnostic performance indicators, and authors’ conclusions.

For studies evaluating circulating kisspeptin, extracted information included assay type, biomarker concentrations, direction of change, odds ratios, confidence intervals, and diagnostic accuracy measures when available. For tissue-based studies, information regarding tissue type, immunohistochemical or molecular assessment methods, expression patterns, and biological interpretation was extracted.

### 2.6. Data Analysis and Narrative Synthesis

Due to substantial heterogeneity in study design, biological material, assay methodology, gestational age at sampling, and outcome reporting, quantitative pooling of results was considered methodologically inappropriate. Therefore, a formal meta-analysis was not performed.

Instead, a narrative and descriptive synthesis was conducted. The included studies were grouped according to the type of evidence generated: (1) studies evaluating circulating serum or plasma kisspeptin levels and (2) studies assessing placental or choriodecidual KISS1/KISS1R expression. Findings were compared with regard to direction of association, consistency across studies, biological plausibility, and relevance to early pregnancy viability.

Particular attention was given to the concordance between systemic and tissue-level findings, methodological differences between studies, and the potential role of kisspeptin as a precision biomarker within precision medicine and personalized pharmaceutical care frameworks.

### 2.7. Risk of Bias Assessment

Risk of bias was assessed independently by two reviewers according to study design. Observational cohort and case–control studies were evaluated using the Newcastle–Ottawa Scale, focusing on selection of study populations, comparability between groups, and outcome assessment. Studies reporting diagnostic accuracy measures were additionally assessed using the QUADAS-2 tool, with attention to patient selection, index test applicability, and outcome verification.

Overall, the included studies demonstrated low to moderate risk of bias. The most common limitations were heterogeneity in gestational age at sampling, variability in assay platforms and kisspeptin isoforms measured, and limited adjustment for potential confounding variables. Disagreements in risk-of-bias assessment were resolved through discussion and consensus.

### 2.8. Ethical Considerations

This study represents a systematic review of previously published literature and did not involve direct participation of human subjects, collection of identifiable patient data, or intervention procedures. Consequently, ethical approval and informed consent were not required. The review was conducted according to principles of transparency, accurate reporting, reproducibility, and appropriate citation of original sources.

## 3. Results

The included studies comprised both clinical biomarker investigations evaluating circulating kisspeptin levels and human tissue-based studies assessing placental KISS1/KISS1R expression [2,3,4,5,7,8]. These complementary approaches allowed integration of systemic and local biological evidence within a clinically relevant human context. The systematic search and study selection process, summarized in the PRISMA 2020 flow diagram, resulted in the inclusion of 6 studies in the qualitative synthesis. These studies comprised observational cohort, case–control, and diagnostic accuracy designs evaluating circulating kisspeptin levels and/or placental *KISS1/KISS1R* expression in early pregnancy loss compared with viable early pregnancies. Despite heterogeneity in study design, gestational age at sampling, and analytical methods, a consistent pattern emerged across the included evidence, allowing structured synthesis of serum biomarker findings and placental immunohistochemical results. The characteristics of the included studies and their key outcomes are summarized in the following tables.

### 3.1. Characteristics of Included Studies

The main characteristics of the included studies are summarized in Table 1, detailing study design, population, gestational age, biological material, and methods used for kisspeptin assessment [2,3,4,5,7,8].

### 3.2. Serum Kisspeptin Levels in Early Pregnancy Loss

Findings from studies evaluating circulating kisspeptin levels in early pregnancy loss are summarized in Table 2. Both included serum-based studies reported significantly lower maternal circulating kisspeptin concentrations in women who subsequently experienced miscarriage compared with women with viable pregnancies. In a large prospective cohort study, Jayasena et al. demonstrated approximately 60% lower plasma kisspeptin concentrations among pregnancies ending in miscarriage and reported that kisspeptin outperformed hCG in predicting pregnancy loss [3]. Similarly, Sullivan-Pyke et al. found significantly reduced serum kisspeptin-54 levels in non-viable pregnancies and demonstrated excellent diagnostic performance for distinguishing miscarriage from viable pregnancy [4].

Despite differences in assay methodology, gestational age at sampling, and reporting units, both studies consistently identified reduced circulating kisspeptin levels as a marker of early pregnancy loss. However, the number of available serum-based studies remains limited, and therefore the current evidence should be interpreted as promising but preliminary [3,4].

### 3.3. Placental KISS1/KISS1R Expression

Placental and choriodecidual expression of KISS1 and/or KISS1R assessed by immunohistochemistry is summarized in Table 3 [2,5,8]. Most included tissue-based studies reported reduced local kisspeptin signaling in pregnancy loss compared with normal gestation [2,5,7,8].

Colak et al. demonstrated significantly reduced placental KISS1 expression in first-trimester spontaneous abortion specimens compared with gestational age-matched controls, suggesting impaired local trophoblastic signaling during early pregnancy loss [2]. Similar findings were reported by Abdelkareem et al., who observed altered KISS1 and KISS1R immunoreactivity in choriodecidual tissues obtained from recurrent pregnancy loss cases compared with controls [8].

Additional mechanistic evidence was provided by Babwah et al., who highlighted the importance of placental KISS1/KISS1R signaling for normal trophoblast function and placental development [5]. Likewise, Li et al. reported dysregulation of the KISS1/GPR54 pathway in trophoblastic tissues from women with recurrent spontaneous abortion, supporting a potential role of impaired kisspeptin signaling in the pathogenesis of pregnancy loss [7].

Overall, the available tissue-based evidence consistently indicates reduced placental kisspeptin activity in miscarriage and recurrent pregnancy loss, although methodological heterogeneity limits direct quantitative comparison among studies [2,5,7,8].

### 3.4. Integrated Analysis of Serum Biomarkers and Placental Tissue Findings

An overall synthesis of the evidence derived from serum biomarker studies and placental tissue analyses is presented in Table 4, highlighting consistency, strength of association, and relevance for individualized monitoring within a precision medicine and personalized pharmacy framework.

Collectively, the included studies provide convergent evidence that both systemic and local kisspeptin signaling are altered in early pregnancy loss [2,3,4,5,7,8]. Serum-based investigations consistently demonstrated lower maternal kisspeptin concentrations in pregnancies ending in miscarriage [3,4], while tissue-based studies reported reduced or dysregulated placental KISS1/KISS1R expression in spontaneous and recurrent pregnancy loss [2,5,7,8].

These findings support the biological concept that kisspeptin reflects trophoblast function, placental development, and maternal fetal interface integrity [5,7]. The consistency observed across independent populations and different biological specimens strengthens the potential role of kisspeptin as a clinically relevant biomarker for early pregnancy viability assessment [2,3,4,7,8].

Nevertheless, differences in study design, gestational age at assessment, laboratory methodology, biomarker quantification, and outcome definitions introduce substantial heterogeneity across the available literature [2,3,4,7,8]. Consequently, larger prospective studies using standardized protocols are required before kisspeptin can be incorporated into routine clinical practice as a predictive biomarker of miscarriage.

## 4. Discussion

The aim of this systematic review was to synthesize and critically evaluate the available evidence on circulating kisspeptin levels and placental KISS1/KISS1R expression in early pregnancy loss, and to consider their potential relevance within precision medicine and personalized pharmacy frameworks. Overall, the included studies showed a consistent association between reduced maternal serum kisspeptin levels and non-viable early pregnancy. In parallel, tissue-based studies demonstrated altered or reduced placental and choriodecidual KISS1/KISS1R expression in miscarriage and recurrent pregnancy loss.

The first major finding concerns circulating kisspeptin as a potential biomarker of early pregnancy viability. Jayasena et al. reported markedly reduced plasma kisspeptin levels in women who subsequently experienced miscarriage, suggesting that maternal kisspeptin may reflect early placental function before clinical pregnancy failure becomes evident. Similarly, Sullivan-Pyke et al. showed that kisspeptin-54 could discriminate viable from non-viable intrauterine pregnancies, supporting its potential value as a non-invasive biomarker. These findings are consistent with the biological role of kisspeptin as a placentally derived peptide whose circulating levels rise substantially during normal pregnancy.

The second important finding relates to placental and choriodecidual expression. Colak et al. demonstrated reduced placental KISS1 expression in first-trimester spontaneous abortion, while Abdelkareem et al. reported altered KISS1/KISS1R immunoreactivity in recurrent pregnancy loss. These tissue-level findings support the hypothesis that early pregnancy loss may involve local dysregulation of kisspeptin signaling at the maternal–fetal interface. This interpretation is also supported by broader international literature showing that kisspeptin participates in trophoblast invasion, implantation, and placental development.

Taken together, the available evidence suggests that systemic and local kisspeptin alterations may represent complementary, although not interchangeable, indicators of placental dysfunction. Serum kisspeptin reflects systemic maternal–placental endocrine activity, whereas placental immunohistochemical expression reflects local tissue-level regulation. Therefore, the concordance between reduced circulating levels and altered placental expression should be interpreted cautiously but provides biological plausibility for the role of kisspeptin in early pregnancy maintenance.

Compared with traditional markers such as β-hCG, kisspeptin may offer a more specific reflection of placental function rather than pregnancy presence alone. However, the current evidence remains preliminary. The included studies differ in assay methodology, kisspeptin isoforms measured, gestational age at sampling, sample size, and outcome definitions. These limitations prevent direct quantitative pooling and highlight the need for standardized prospective studies before clinical implementation.

Although reduced circulating kisspeptin levels and decreased placental KISS1/KISS1R expression may point in the same biological direction, these domains should not be considered directly interchangeable. Circulating levels reflect systemic dynamics, whereas tissue expression reflects local regulation at the maternal–fetal interface. Therefore, concordance should be interpreted cautiously.

From the perspective of personalized pharmacy, the relevance of kisspeptin lies primarily in its potential role within individualized monitoring frameworks rather than immediate therapeutic decision-making. Pharmacists may contribute through biomarker-informed counseling, interpretation support, and coordination within multidisciplinary care. At present, however, this role remains prospective rather than established in routine practice.

### 4.1. Maternal Serum Kisspeptin as a Biomarker of Early Pregnancy Viability

One of the most consistent findings across the included studies is the significant reduction in maternal serum kisspeptin levels in early pregnancy loss compared with viable pregnancies. As summarized in Table 2, both large prospective cohorts and case–control studies demonstrated a strong inverse association between circulating kisspeptin concentrations and miscarriage risk. Kisspeptin levels measured during the first trimester were shown to be markedly lower—by approximately 60%—in women who subsequently miscarried, with high discriminatory accuracy and odds ratios indicating a substantial protective association with ongoing pregnancy.

These findings are biologically plausible given that placental synthesis is the predominant source of circulating kisspeptin during pregnancy. Unlike traditional biomarkers such as β-hCG, which primarily reflect trophoblastic mass and endocrine activity, kisspeptin appears to reflect broader aspects of placental function, including trophoblast differentiation and invasion. The superior predictive performance of kisspeptin compared with hCG reported in several studies suggests that kisspeptin may serve as a more sensitive early indicator of placental dysfunction, rather than merely pregnancy presence.

### 4.2. Placental Kisspeptin Expression and Local Dysregulation

Beyond systemic alterations, the present review highlights consistent evidence of disrupted placental kisspeptin signaling at the tissue level. Immunohistochemical analyses demonstrated reduced expression of KISS1 and its receptor KISS1R in placental and choriodecidual tissues from early pregnancy loss and recurrent pregnancy loss cases (Table 3). These findings support the concept that local kisspeptin deficiency may contribute directly to abnormal placentation.

Kisspeptin has been shown to regulate trophoblast migration and invasion in a tightly controlled, dose-dependent manner. Reduced expression of KISS1/KISS1R in miscarriage placentas is therefore consistent with impaired trophoblast invasion, shallow placentation, and subsequent pregnancy failure. The concordance between reduced circulating kisspeptin levels and diminished placental expression strengthens the argument that systemic measurements may reflect underlying local placental pathology.

### 4.3. Integration of Serum and Tissue-Level Findings

A key strength of this review lies in the integration of serum biomarker data with placental immunohistochemical findings. While many previous studies have focused exclusively on circulating kisspeptin concentrations, the combined evaluation of systemic and local expression provides a more comprehensive understanding of kisspeptin’s role in early pregnancy maintenance. As summarized in Table 4, evidence across both domains demonstrates moderate to high consistency and strong biological plausibility.

Importantly, the observed alterations in kisspeptin signaling are not merely associative but are supported by mechanistic data from experimental and animal studies. Downregulation of KISS1/KISS1R has been linked to altered trophoblast behavior, reduced invasiveness, and defective placental development, providing a mechanistic bridge between molecular findings and clinical outcomes.

### 4.4. Comparison with Other Pregnancy Complications

Interestingly, kisspeptin dysregulation is not unique to early pregnancy loss. In disorders such as preeclampsia, studies have reported a paradoxical pattern characterized by reduced circulating kisspeptin levels alongside increased placental expression. This divergence underscores the complexity of kisspeptin regulation at the maternal–fetal interface and suggests that systemic and local expression may be differentially regulated depending on the underlying placental pathology. Such findings further support the need to interpret circulating kisspeptin levels within the broader context of placental biology rather than as an isolated biomarker.

### 4.5. Clinical Implications

From a clinical perspective, the findings of this review suggest that maternal serum kisspeptin holds promise as a non-invasive biomarker for early pregnancy viability. Its potential advantages over existing markers include higher discriminatory accuracy and closer reflection of placental function. Furthermore, the integration of serum kisspeptin measurements with placental biomarkers or traditional hormonal assays may enhance early risk stratification for pregnancy loss.

However, before kisspeptin can be incorporated into routine clinical practice, several challenges must be addressed. These include standardization of assay methodology, determination of gestational age–specific reference ranges, and validation in large, diverse prospective cohorts. Without such standardization, inter-study variability may limit clinical applicability.

### 4.6. Strengths and Limitations

A key strength of this systematic review is the integration of circulating biomarker data with placental tissue-level evidence, allowing a multi-level precision medicine perspective on kisspeptin dysregulation in early pregnancy loss. By adhering strictly to PRISMA 2020 guidelines and predefined inclusion criteria, the review avoids overinterpretation and provides a transparent synthesis of available evidence relevant to personalized pharmaceutical monitoring.

Several limitations should be acknowledged. First, the number of clinical studies, particularly those evaluating circulating kisspeptin, remains limited. Second, substantial heterogeneity exists across studies in terms of gestational age, assay methodology, and reporting formats. Third, most studies are observational, limiting causal inference. Finally, differences between circulating and tissue-based findings further complicate interpretation. Therefore, current evidence should be considered preliminary and requires further validation.

### 4.7. Future Directions

Future research should focus on large-scale prospective studies combining serial serum kisspeptin measurements with detailed placental phenotyping. Standardized immunohistochemical scoring systems and harmonized laboratory assays would further strengthen comparability across studies. Additionally, integrating kisspeptin into multimarker models alongside established biochemical and ultrasound parameters may improve early prediction and mechanistic understanding of pregnancy loss.

## 5. Conclusions

This systematic review indicates that reduced maternal serum kisspeptin levels and altered placental or choriodecidual KISS1/KISS1R expression are consistently associated with early pregnancy loss across the available evidence. These findings support the biological plausibility of kisspeptin as a candidate biomarker reflecting both systemic and local placental dysfunction in early gestation.

From a clinical perspective, kisspeptin may have future value as part of individualized monitoring strategies for women at risk of early pregnancy loss, particularly when combined with established biochemical and ultrasound parameters. Its potential role may be especially relevant in early risk stratification, patient counseling, and multidisciplinary follow-up planning. However, kisspeptin should not yet be considered a clinically validated diagnostic marker because of the limited number of studies, heterogeneity in assay methods, and lack of standardized gestational age-specific reference ranges.

From a policy and healthcare implementation perspective, future integration of kisspeptin testing would require standardized laboratory protocols, validated clinical cut-off values, cost-effectiveness evaluation, and clear guidance for interpretation within obstetric and pharmaceutical care pathways. Pharmacists and other healthcare professionals may contribute to biomarker-informed counseling and patient-specific monitoring once sufficient clinical validation is available.

Further large-scale, multicenter prospective studies are required to confirm the diagnostic and prognostic value of kisspeptin, harmonize measurement methods, and determine whether its use improves clinical decision-making and pregnancy outcomes.

## Figures and Tables

**Table 1:** Characteristics of included studies.

Author (Year)	Country	Study Design	Population	Sample Size (EPL/RPL)	Sample Size (Controls)	Gestational Age (Weeks)	Biomarker Assessed	Biological Material	Method
Jayasena et al. (2014) [3]	UK	Prospective cohort	Asymptomatic early pregnancy	102 EPL	993 ongoing	6–10	Kisspeptin	Maternal plasma	ELISA
Sullivan-Pyke et al. (2018) [4]	USA	Case–control	Viable vs. non-viable pregnancy	69 miscarriage	44 viable	5–9	Kisspeptin-54	Maternal plasma	ELISA
Colak et al. (2020) [2]	Turkey	Case–control	First trimester pregnancy	30 abortion	30 controls	6–12	KISS1	Placental tissue	IHC
Babwah et al. (2015) [5]	Canada	Human tissue-based study/observational	Early pregnancy placentas	NR	NR	First trimester	KISS1/KISS1R	Placental tissue	IHC/molecular
Li et al. (2017) [7]	China	Translational human tissue study RSA model	Recurrent spontaneous abortion	RSA model	Controls	First-trimester equivalent	KISS1/KISS1R	Trophoblast tissue	IHC/PCR
Abdelkareem et al. (2023) [8]	USA	Case–control	Recurrent pregnancy loss	25 RPL	20 controls	First trimester	KISS1/KISS1R	Choriodecidual tissue	IHC

**Table 2:** Studies evaluating circulating kisspeptin (subset of included studies).

Author (Year)	Gestational Age	EPL/Miscarriage Value	Control Value	Unit	Direction of Change	Statistical Significance	Diagnostic Performance	Authors’ Conclusion
Jayasena et al. (2014) [3]	6–10 weeks	~60% lower	Reference	nmol/L	Decreased in EPL	*p* < 0.001	OR 0.13 (95% CI 0.08–0.22)	Kisspeptin superior to hCG in predicting miscarriage
Sullivan-Pyke et al. (2018) [4]	5–9 weeks	Significantly reduced	Higher in viable	ng/mL	Decreased in EPL	*p* < 0.01	AUC ~0.90	Kisspeptin discriminates viable vs. non-viable pregnancy

**Table 3:** Studies evaluating placental KISS1/KISS1R expression (subset of included studies).

Author (Year)	Tissue Type	Gestational Age	Marker	Scoring System	Expression in EPL/RPL	Expression in Controls	Statistical Significance	Interpretation
Colak et al. (2020) [2]	Placenta	6–12 weeks	KISS1	Semi-quantitative IHC	Reduced expression	Higher expression	*p* < 0.05	Impaired local kisspeptin signaling in abortion
Babwah et al. (2015) [5]	Placenta	First trimester	KISS1/KISS1R	Semi-quantitative IHC	Downregulated	Normal expression	Significant	Defective trophoblast invasion
Abdelkareem et al. (2023) [8]	Choriodecidua	First trimester	KISS1/KISS1R	IHC	Altered/reduced	Preserved	*p* < 0.05	Local dysregulation in RPL

**Table 4:** Evidence Synthesis.

Domain	Number of Studies	Consistency of Findings	Strength of Association	Overall Level of Evidence
Serum kisspeptin and early pregnancy loss	limited number of studies	High	Suggestive inverse association	Moderate–high
Placental *KISS1/KISS1R* expression (immunohistochemistry)	available evidence	Preliminary to moderate evidence	Consistent reduction in EPL/RPL	Moderate

## Data Availability

No new data were created or analyzed in this study. Data sharing is not applicable to this article, as it is based exclusively on previously published studies.
